# Standardised proformas improve patient handover: Audit of trauma handover practice

**DOI:** 10.1186/1754-9493-2-24

**Published:** 2008-09-25

**Authors:** Nicholas A Ferran, Andrew J Metcalfe, Declan O'Doherty

**Affiliations:** 1 Department of Trauma and Orthopaedics, University Hospital of Wales, Cardiff, UK

## Abstract

**Background:**

The implementation of the European Working Time Directive has meant the introduction of shift patterns of working for junior doctors. Patient handover between shifts has become a necessary part of practice in order to reduce the risk of medical errors. Data handed over between shifts are used to prioritise clinical jobs outstanding, and to create theatre lists. We present a closed-loop audit of handover practice to assess whether standardised proformas improve clinical data transfer between shifts during handover in our Orthopaedic Unit.

**Methods:**

We collected data handed over between shifts for a period of one week at our department. The data were in the form of hand written data on plain paper used to assist verbal handover. Data were analysed and a standardised handover sheet was trialled. After feedback from juniors the sheet was revised and implemented. A re-audit, of handover data, was then undertaken using the revised standardised proforma during a period of 1 week.

**Results:**

Forty-eight patients were handed over in week 1 while 55 patients were handed over during re-audit. The standardised proformas encouraged use of pre-printed patient labels which contained legible patient identifiers, use of labels increased from 72.9% to 93.4%. Handover of outstanding jobs increased from 31.25% to 100%. Overall data handed over increased from 72.6% to 93.2%. Handover of relevant blood results showed little improvement from 18.8% to 20.7%

**Conclusion:**

This audit highlights the issue of data transfer between shifts. Standardised proformas encourage filling of relevant fields and increases the data transferred between shifts thereby reducing the potential for clinical error cause by shift patterns.

## Background

In several countries around the globe there have been efforts to reduce the working hours of junior doctors. In the UK the introduction of the European Working Time Directive has led to the introduction of shift patterns of working and as hours are reduced the numbers of shifts are increased. As shift patterns increase, continuity of care decreases [1]. In New Zealand it has been estimated that the average medical patient sees 6 doctors per admission and the average surgical patient sees 10 [2]. A survey of handover practice in Wales concluded that there was no standardisation of handover practice and only 2 of 17 hospitals used a standardised handover proforma. In 13 of 17 hospitals personal lists on blank pages were commonly used [3]. Verbal handover is prone to data loss which can compromise patient care [4]. The British Medical Association in its guidance on clinical handover "Safe Handover – Safe Patients" recommends the use of standardised proformas and relevant IT support for clinical handover [5]. The Royal College of Surgeons of England in its guidance "Safe Handover" set out minimum data necessary for safe handover [6]. We present the results of an audit into handover practice that demonstrates the efficacy of standardised handover proformas.

## Methods

### Handover practice

In our department handover occurs between shifts twice per day. At 20:00 hours handover between the junior day and night teams occurs. Patients admitted throughout the preceding shift are handed over to the night team who are unfamiliar to the admitted patient. No senior surgeons (consultants) attend this handover. At 08:00 hours handover occurs between the on-call team from the previous day and night shift to the senior surgeon (consultant) on-call. Data recorded on handover sheets are used to prompt the handover sessions and the sheets are passed between shifts.

### Methodology

Orthopaedic admissions handed over during a one week period in a teaching hospital were collected. Patient data were handed over by the on-call doctor for Trauma & Orthopaedics. The data were hand written on plain paper and used to assist verbal handover. The data were analysed and essential information necessary for safe patient handover was identified. A standardised proforma was designed to assist in handover and encourage documentation of this essential dataset in accordance with Royal College of Surgeons of England guidelines [6].

The first version of the handover proforma was piloted over a 3 week period and reassessed. There was 78% compliance with version 1 of the proforma. The results of this pilot study have been published [7]. Feedback was obtained from the junior doctors using the forms and the proforma was redesigned to include aspects that made use of the forms during shifts practical such as including space for ward locations of the patients which allowed them to better organise ward rounds. Version 2 of the handover form [see Additional file 1] was then implemented.

A re-audit, of handover data, was then undertaken using the revised standardised handover sheets during a period of 1 week. The gold standard for audit was 100% data transmission as suggested by Bhabra et al. [4] and 100% compliance with standardised proformas.

Each item of data was classified as either present or missing. Blood tests were only considered relevant where they might be expected to impact on a patient's management (i.e. bloods results were considered relevant for someone with a potentially septic joint but not for a young patient with a tendon laceration). Statistical comparisons of individual types of data before and after the introduction of the proformas were performed using Fisher's exact test.

For each patient a score out of seven (or eight if relevant bloods tests were required) was given for the total amount of data handed over per patient. For example, a score of 7 out of 7 indicated that each item had been handed over, whereas a score of five out of eight would indicate that three items (e.g. hospital number and side) were missing from handover. For statistical analysis, these were expressed as percentages.

The Mann-Whitney U test was used to compare the total amount of data handed over for each patient before and after the introduction of the proformas. The calculations were performed using SPSS version 12 (SPSS Inc. Chicago, Illinois, USA). A level of p < 0.05 was taken to indicate statistical significance.

## Results

During first week of audit, 48 patients were handed over using handwritten sheets. Fifty-five patients were handed over during a one week period with the standardised proformas (version 2) and there was 100% compliance with the new proforma.

**Figure 1:**
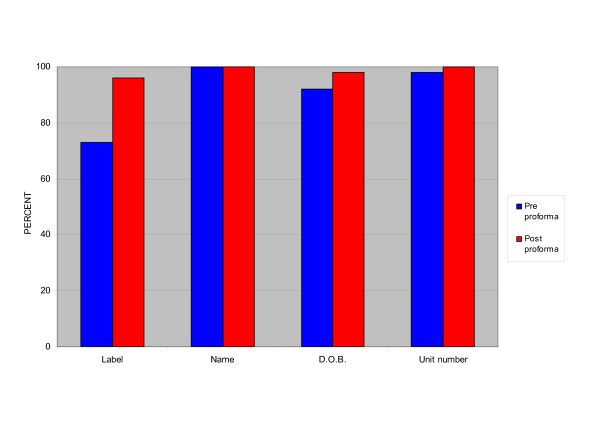
Bar chart of percentage of patient data transferred pre/post proforma.

Of the initial 48 patients handed over, a computer generated patient identifier label was used in only 35 cases (73%), all other patients were handed over with hand-written identifiers. In all cases where labels were not used the patient name was handed over but in 4 patients (8%) no date of birth was handed over and in 1 patient (2%) no patient unit number was handed over. Use of patient labels increased to 53 patients (96%) with the new sheets and the date of birth was missing in one (2%) (Figure 1).

On the plain paper sheets, the diagnosis was not handed over in 4 patients (8%) and relevant side of injury was omitted in 20 patients (42%). Tasks performed or outstanding were only handed over in 15 patients (31%) and relevant blood results were only handed over in 6 of 32 patients (19%).

**Figure 2:**
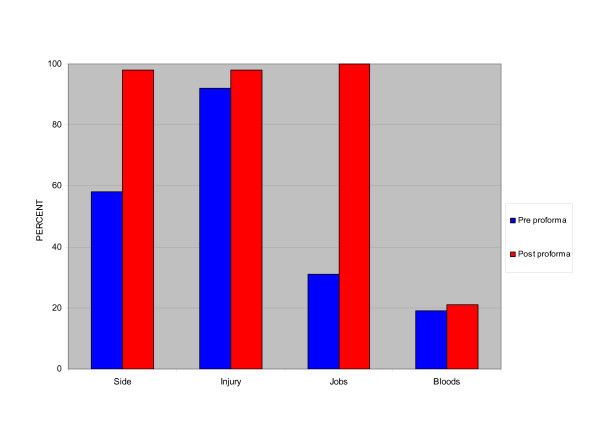
Bar chart of percentage of patient data transferred pre/post proforma.

Analysis of handover using the standardised proformas (version 2) demonstrated an improvement in the hand over of diagnosis and the side of injury, which were only missed once for each category (98% compliance for both). Tasks performed or outstanding were handed over in all cases (100%), however the handover of relevant blood results remained poor: 19% without the proforma and 20% with the proforma. Overall data transfer improved from 73% with the hand-written sheets to 93% with the use of standardised proformas (Figure 2).

The improvement in data handed over after the new sheets were introduced was significant for the use of pre-printed labels, the side of injury and the tasks performed or outstanding (all at p < 0.01) (Table 1). The difference in overall data transferred for each patient was highly significant (p < 0.01).

**Table 1:** Data handed over by data type.

	Handwritten sheets (n = 49)		Handover proforma (n = 55)		Significance
	Handed over	Missing	Handed over	Missing	

Label	35	14	53	2	p < 0.01
Name	48	1	55	0	N.S.
Date of Birth	44	5	54	1	N.S (p = 0.13)
Unit number	47	2	55	0	N.S (p = 0.28)
Side of injury	28	21	54	1	p < 0.01
Injury	44	5	55	0	N.S (p = 0.13)
Tasks performed or outstanding	15	34	55	0	p < 0.01
Relevant blood results (pre n = 32; post n = 29)	6	26	6	23	N.S. (p = 0.84)
Total data handed over per patient	72.6% (S.D. 16.0%)		93.2% (S.D. 9.6%)		p < 0.01

## Discussion

Patient data handed over between shifts are essential to continuity of care. In the case of trauma patients, the handover data are also used to prepare trauma lists. The accuracy of patient identifiers and the side and site of intended surgery is crucial to the avoidance of wrong side surgery. Our study demonstrated that the use of a structured handover proforma increased data transfer from 73% to 93%. The proformas developed encouraged the use of computer generated patient identifier labels thereby ensuring the accuracy of patient identifiers. Use of the proformas also improved recording of patient diagnosis, side of injury and handover of tasks performed or outstanding.

The main limitations to the study were that handover sessions were not witnessed by the authors, and only data recorded on handover sheets or proformas were considered as having been handed over. This approach was adopted as it was felt that attending or recording handover sessions would change practice of the doctors involved and thus introduce bias. Doctors involved in the handover sessions were told that the collection of handover sheets was to trial a database and therefore had no knowledge that their practice was under review. It has already been demonstrated in simulated handover that verbal handover is unreliable and prone to data loss [4], and as a result we considered only data recorded on handover sheets or proformas as having been handed over as this data would be less likely to be forgotten or overlooked.

McCann et al. found that 60.9% of doctors had experience clinical problems related to poor handover in a New Zealand tertiary hospital. In their survey, 31% of doctors indicated that an 'on-call'/handover sheet would most effectively improve patient handover [8]. In the United States, 31% of doctors surveyed experienced clinical problems during their shift that they could have been prepared for with adequate handover [1]. These studies highlight that handover is often inadequate.

In an experimental evaluation of handover practice Bhabra et al. reported only 33% data transfer with verbal handover, verbal handover with note taking improved data transfer to 92%. In their study a computer generated, pre-printed handover sheet improved data transfer to 100%. This involved doctors entering data into computers continuously during their shifts [4].

Simplicity is key to effective handover and the use of IT systems for support of the handover process must be practical [8]. While computer generated handover sheets can provide 100% data transfer, they rely on data entry throughout a shift and accessible IT stations throughout the workplace. In a busy tertiary centre such as ours, where patients may be spread over many wards, compliance with such a process may be poor and may lead to inefficiency. Ensuring that there are accessible computer terminals throughout the workplace also comes at a significant cost. Technological solutions are also no substitute for effective communication [1].

The handover proforma developed in this study encourage the use of computer generated patient identifier labels. These labels increase the accuracy of transfer of patient identifiers which is essential to the creation of theatre lists. Responding to the feedback of the doctors involved and adapting the forms to assist in the efficiency of their shift work such as ward rounds, had resulted in 100% compliance during the audit period. The handover of tasks performed and outstanding has improved to 100% and therefore improves the continuity of care, however, the handover of relevant blood results remains poor and is of concern. We believe that this may be because there is not a specific field on the form for entering blood results thereby acting as a prompt. The authors are considering making further amendments to the form to encourage the handover of blood results bearing in mind that the forms must not become so cumbersome as to affect compliance.

## Conclusion

The introduction of shift patterns of working, in order to reduce the working hours of junior doctors, has affected the working practice of the medical profession the world over. Surgeons continue to adapt and develop their working practice in order to deliver excellence in clinical care despite increasing shift patterns, which challenge continuity of care. Patient handover continues to be a weak link in the delivery of care. The development of standardised handover proformas can improve the efficiency of data transfer during patient handover.

## Competing interests

The authors declare that they have no competing interests.

## Authors' contributions

NAF & AJM were responsible for study design, proforma design, data collection and analysis. NAF prepared the manuscript. AJM & DOD were responsible for manuscript editing and review. All authors have read and approved the final manuscript.

## Supplementary Material

Additional file 1 Handover proforma version 2. This was the proforma designed to encourage handover of patient data. Click here for file
