# Supplementary material for: Standardised proformas improve patient handover: Audit of trauma handover practice
Source: Patient Saf Surg. 2008 Sep 25;2:24. doi: 10.1186/1754-9493-2-24 (PMC2565654; doi:10.1186/1754-9493-2-24)
Supplement: Additional file 1 — Handover proforma version 2. This was the proforma designed to encourage handover of patient data. [file 1754-9493-2-24-S1.doc]

**APPENDIX 1** HANDOVER SHEET

| REF BY | PATIENT INFORMATION | DIAGNOSIS | OUTSTANDING JOBS / RESULTS |
| --- | --- | --- | --- |
| A&E 🗆  GP 🗆  #CL 🗆  TRA 🗆  WD 🗆 | AFFIX LABEL HERE | L 🗆 R 🗆 BILAT 🗆 | CLERK 🗆  BLOODS 🗆  XRAY 🗆  CHECK XRAY 🗆  CON. & MRK 🗆  TH. CARD 🗆  REG RV 🗆 |
| WARD |
| A&E 🗆  GP 🗆  #CL 🗆  TRA 🗆  WD 🗆 | AFFIX LABEL HERE | L 🗆 R 🗆 BILAT 🗆 | CLERK 🗆  BLOODS 🗆  XRAY 🗆  CHECK XRAY 🗆  CON. & MRK 🗆  TH. CARD 🗆  REG RV 🗆 |
| WARD |
| A&E 🗆  GP 🗆  #CL 🗆  TRA 🗆  WD 🗆 | AFFIX LABEL HERE | L 🗆 R 🗆 BILAT 🗆 | CLERK 🗆  BLOODS 🗆  XRAY 🗆  CHECK XRAY 🗆  CON. & MRK 🗆  TH. CARD 🗆  REG RV 🗆 |
| WARD |
| A&E 🗆  GP 🗆  #CL 🗆  TRA 🗆  WD 🗆 | AFFIX LABEL HERE | L 🗆 R 🗆 BILAT 🗆 | CLERK 🗆  BLOODS 🗆  XRAY 🗆  CHECK XRAY 🗆  CON. & MRK 🗆  TH. CARD 🗆  REG RV 🗆 |
| WARD |
| A&E 🗆  GP 🗆  #CL 🗆  TRA 🗆  WD 🗆 | AFFIX LABEL HERE | L 🗆 R 🗆 BILAT 🗆 | CLERK 🗆  BLOODS 🗆  XRAY 🗆  CHECK XRAY 🗆  CON. & MRK 🗆  TH. CARD 🗆  REG RV 🗆 |
| WARD |
| A&E 🗆  GP 🗆  #CL 🗆  TRA 🗆  WD 🗆 | AFFIX LABEL HERE | L 🗆 R 🗆 BILAT 🗆 | CLERK 🗆  BLOODS 🗆  XRAY 🗆  CHECK XRAY 🗆  CON. & MRK 🗆  TH. CARD 🗆  REG RV 🗆 |
| WARD |

CONSULTANT ON CALL_____________ DATE_____________
